# How to create coats for all seasons: elucidating antigenic variation in African trypanosomes

**DOI:** 10.1042/ETLS20170105

**Published:** 2017-12-22

**Authors:** Cher-Pheng Ooi, Gloria Rudenko

**Affiliations:** Department of Life Sciences, Sir Alexander Fleming Building, Imperial College London, South Kensington, London SW7 2AZ, U.K.

**Keywords:** African trypanosome, antigenic variation, epigenetics, monoallelic exclusion, variant antigen genes, variant surface glycoprotein

## Abstract

Extracellular parasites of the mammalian bloodstream face considerable challenges including incessant assault by the immune system. African trypanosomes are consummate survivors in this inclement environment and are renowned for their supremely sophisticated strategy of antigenic variation of their protective surface coat during the course of chronic infections. Recent developments are making us realize how complex this antigenic machinery is and are allowing us to tackle previously intractable problems. However, many of the simplest (and arguably the most important) questions still remain unanswered!

## How is the extraordinary heterogeneity of variant surface glycoprotein variants during a chronic infection generated?

The bloodstream form of *Trypanosoma brucei* is coated with a dense covering of ∼10^7^ variant surface glycoprotein (VSG) molecules, which extend as antigenically variable rods connected to the cell surface via glycosylphosphatidylinositol (GPI) anchors [1]. This GPI attachment allows VSGs to move freely over the cell surface [2]. Tight packing of these VSGs produces a protective barrier shielding invariant surface proteins from recognition by host antibodies and preventing trypanosome lysis by the alternative pathway of the complement system [3]. Extraordinarily high rates of recycling of surface VSG allow selective removal of host cell molecules including antibodies and complement from the trypanosome surface, thereby functioning as a ‘coat-cleaning’ machine [4]. This prolongs trypanosome survival in rising antibody titres and facilitates escape from macrophages [5].

**Figure 1.**
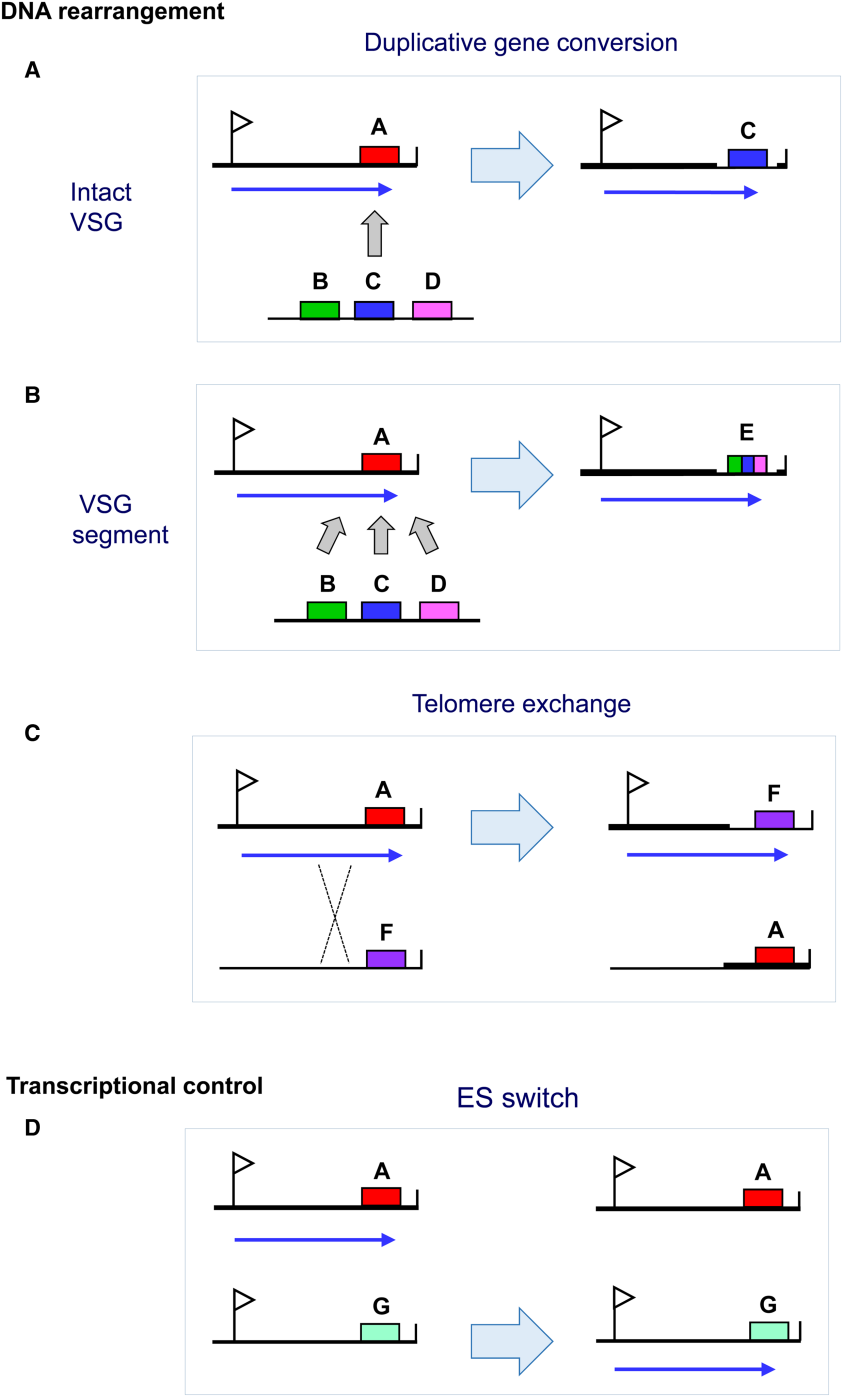
Mechanisms for switching VSG. VSG switching can be mediated through DNA rearrangements (gene conversion or telomere exchange) or transcriptional control. Duplicative gene conversion involves copying an intact *VSG* (**A**) or segments of a *VSG* (**B**) into the active *VSG* ES resulting in loss of the old *VSG.* After a telomere exchange (**C**), there is no loss of genetic information. A transcriptional switch entails activating a new ES and silencing the old one (**D**). ESs are shown with the promoters indicated with flags, *VSG* genes with coloured boxes, and transcription of the active ES with an arrow.

Each trypanosome has a vast repertoire of *VSG* genes [6], differing in number and content between different strains, presumably as a consequence of diversifying forces. For example, the ‘VSGnome’ of *T. brucei* 427 contains more than 2500 different *VSG* genes, of which more than 80% are not immediately functional [7]. Switching the active VSG can entail transcriptional switches between the ∼15 telomeric *VSG* expression site (ES) transcription units (Figure 1). More importantly, DNA rearrangements and predominantly gene conversions can copy new *VSG*s (or segments of *VSG*s) into the active ES, thereby resulting in an antigenic switch [8] (Figure 1). This extraordinary ability to construct novel patchwork, or mosaic, *VSG*s is key for trypanosome survival [9]. The continuous recombination of various permutations and combinations of sections of *VSG* genes (or pseudogenes) allows the trypanosome to construct an almost infinite number of antigenically distinct VSG coat types.

A given wave of trypanosome parasitaemia is normally predominantly composed of a major VSG variant, although it has long been known that minor VSG variants are also present within each wave. However, there is possibly phenomenal heterogeneity within these infection waves, as next-generation RNA sequencing of RNA transcripts has documented an extraordinary variety of minor VSG variants [10]. These experiments argue that a considerable percentage of the available *VSG* repertoire is displayed relatively early in the trypanosome infection. This highlights the necessity of the continuous generation of mosaic *VSG*s for long-term trypanosome survival. However, this also gives rise to several questions. Where are these new *VSG*s made? Within a silent ES? How then does the trypanosome ‘test’ that a new *VSG* cobbled together from different pieces is indeed functional? Within an active ES? However, VSG is essential for the bloodstream-form trypanosome, and blocking its synthesis triggers a precytokinesis arrest [11]. This does not give much time for multiple successive gene conversions to occur, unless this process is transiently stimulated. What is the DNA recombination machinery that allows the trypanosome to construct these novel *VSG* mosaics? Is this specifically dedicated to the generation of *VSG* diversity?

Key questions remain unanswered. However, a major complication with analyzing VSG switching in chronic infections is that establishing the order of VSG switching is often not possible. Major variants following each other in successive waves of parasitaemia might not necessarily have been generated from each other, but can be the consequence of the emergence of minor variants that have arisen much earlier. Unravelling this complexity will be seriously non-trivial.

## How is VSG expression controlled in a monoallelic fashion?

To allow frugal use of the *VSG* repertoire, only a single *VSG* is highly expressed per cell from one of ∼15 highly similar telomeric *VSG* ESs [12]. ES transcription is mediated by RNA polymerase I (Pol I), which also transcribes the ribosomal DNA [13]. The use of Pol I to transcribe protein-coding genes is unprecedented in eukaryotes, where protein-coding genes are normally exclusively transcribed by Pol II. The use of an unconventional RNA polymerase makes it likely that ES control will involve some very novel features, which yet remain to be unravelled. The active ES is located in a unique non-nucleolar Pol I-containing structure called the expression site body (ESB) [14]. Low levels of transcripts are generated from ‘silent’ ESs which are not located in the ESB [15], but these RNAs are predominantly non-polyadenylated [16]. This could argue that the ESB is a ‘factory’ which contains components allowing efficient transcription initiation and elongation at the active ES, as well as RNA processing including trans-splicing and polyadenylation. The ESB appears to be Pol I-transcription nucleated, as it disappears exceptionally rapidly after the specific blocking of Pol I transcription using chemical Pol I inhibitors [17].

Whole-genome RNAi libraries are now allowing the development of functional screens to identify essential components involved in basic biochemical processes in African trypanosomes [18]. A whole-genome RNAi screen designed to select for derepression of a silent *T. brucei* telomere has allowed the identification of the first ESB-specific marker, a protein called VEX1 [19]. The identification of additional ESB-specific components, as well as determination of their respective functions, should give us insights into the functioning of this unique subnuclear body.

A single trypanosome transcribes a single ES at a time, with the remaining 14 ESs remaining essentially inactive. What mediates this singularity of ES expression [20]? Using drug selection pressure, it is possible to force the trypanosome to simultaneously activate a second ES; however, this state is not well tolerated by the cell. The resulting ‘double-expresser’ state is highly unstable, and the trypanosomes appear to rapidly switch back and forth between the two ESs [21]. More detailed analysis of these ‘double-expresser’ strains should give us insights into the epigenetic modifications which facilitate this perturbation of the monoallelic exclusion machinery. However, central questions regarding the molecular restrictions mediating this striking singular ES expression still remain unanswered.

## How do epigenetic factors affect *VSG* expression and switching?

DNA in eukaryotes is wrapped around nucleosomes composed of the core histones, with chromatin remodelling allowing the cell to modulate the accessibility of DNA regulatory sequences to the transcriptional machinery. Specific post-translational modifications of the histones, particularly at their N-termini, allow the cell to functionally define different types of chromatin. African trypanosomes do not appear to regulate their Pol II transcription, and the bulk of the genome is constitutively transcribed in extensive polycistronic transcription units [22]. This has led to a relatively late appreciation of the role of chromatin in the organization and transcription of the *T. brucei* genome.

Next-generation sequencing is now allowing the investigation of nucleosome positioning in unprecedented detail across the entire *T. brucei* genome. The position of individual nucleosomes across all of the core chromosomes has now been determined in both bloodstream- and procyclic-form *T. brucei* [23,24]. This has allowed us to determine the chromatin state at different types of transcription units. Pol II promoters in *T. brucei* are diffuse and lack distinctive sequence elements [25]. Instead, Pol II promoters appear to be predominantly functionally defined by discrete chromatin structure around the strand switch regions where Pol II transcription initiates [24]. In addition, chromatin structure appears to play a role in the repression of the silent *VSG* genes, and positioned nucleosomes are found flanking the silent *VSG* gene arrays [23]. The chromatin structure in the border regions of these *VSG* arrays could represent barrier regions which repress cryptic transcription initiation or readthrough into the *VSG* arrays, keeping these *VSGs* silent.

**Figure 2.**
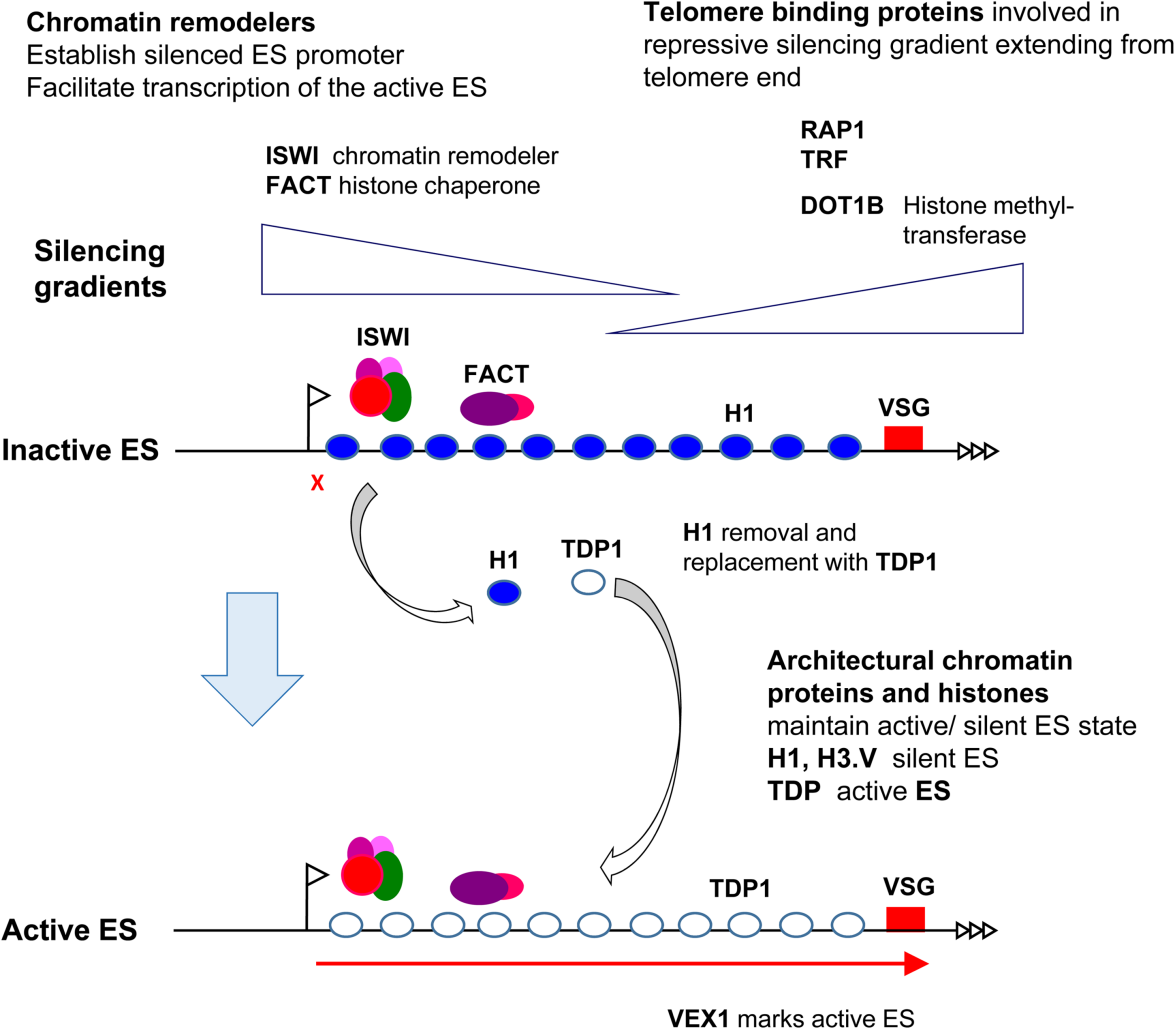
Schematic of *VSG* expression site control mediated via opposing repressive chromatin gradients. ESs in different activation states are shown with flags indicating the promoters, transcription with a red arrow and telomere repeats with arrow heads. Chromatin proteins and remodellers (indicated with coloured circles) help maintain a silenced chromatin state at inactive ES promoters. Telomere-binding proteins including RAP1 and TRF help maintain a repressed state at the chromosome end. These operate in a gradient extending upwards from the chromosome end, in combination with the histone methyltransferase DOT1B. The linker histone H1 coats inactivate ESs (blue circles), but upon ES activation is replaced with TDP1 (open balls), which maintains an open chromatin state. VEX1 is a marker for the active ES.

In contrast, the active ES has an unusually open chromatin structure compared with most of the *T. brucei* genome and is stripped bare of most nucleosomes, as well as the linker histone H1 (Figure 2) [26–28]. This strikingly open chromatin state is characteristic of Pol I-transcription units and presumably facilitates the exceptionally high rate of transcription initiation seen here [29]. In place of nucleosomes, the architectural chromatin protein TDP1 coats the active *VSG* ES [30]. TDP1 is a high mobility group protein which is likely to be analogous to HMO1 in yeast [31]. HMO1 coats active Pol I-transcription units with a mutually exclusive distribution to the linker histone H1 and appears to stabilize chromatin depleted of nucleosomes [29,32]. Consistent with this, in *T. brucei*, TDP1 appears to help maintain an active chromatin state at the ES, even if ES transcription itself is inducibly blocked [33].

In contrast, silent ESs are marked with an unusual modified glucosylated nucleotide called Base J, which is incorporated in a gradient that increases towards the telomere end and could play some role in silencing these telomeric *VSGs* [34,35]. Additionally, different histone variants and modifications have also been shown to play a role in the down-regulation of ESs (Figure 2). The histone variant H3.V is enriched around the termination region of Pol II transcription units [25]. Knockdown of histone variant H3.V not only results in the disruption of Pol II transcription termination but also causes the derepression of the silent telomeric ESs [34,35]. Histone modifications also play an important role in ES control. Knockdown of the H3K76 histone methyltransferase DOT1B results in derepression of silent ESs and slows down the kinetics of transcriptional switching between ESs [36]. However, histone modifications, which are unique for ESs, have yet to be identified. Mass spectrometry analysis of *T. brucei* histones should allow us to identify additional modifications which could prove to be crucial for understanding epigenetic control.

It is now clear that a wide range of epigenetic factors play a role in the control of *VSG* expression, and a long list of chromatin remodellers, histone-modifying enzymes, chromatin ‘readers’ of histone modifications and chromatin architectural proteins have been shown to be important [37]. It is vital that more are identified, as the connections between these different puzzle pieces still remain to be elucidated.

## How does repressed chromatin suppress *VSG* switching mediated through DNA recombination?

ESs are extensive telomeric transcription units, normally ranging 40–60 kb [12]. Repressive chromatin domains or gradients appear to control ESs from both ends [20]. An inhibitory chromatin state appears to restrict transcription initiation and elongation at *VSG* ES promoters, as knockdown of chromatin remodellers including ISWI and FACT results in derepression of silent ESs [38,39]. In addition, a repressive chromatin gradient mediated by the chromatin protein RAP1 extends up from the telomere end, and knockdown of RAP1 results in derepression of silent ESs [40].

However, in addition to having an impact on transcription, repressed chromatin states also appear to suppress excessively high rates of DNA recombination at the ES telomere. RAP1 knockdown results not only in ES depression but also in an increase in double-strand DNA breaks at the ES telomere, with a concurrent increase in *VSG* switching mediated by DNA recombination [41]. Intriguingly, this appears to occur through transcriptional regulation of a non-coding RNA. Knockdown of RAP1 results in the depression of transcription of the telomeric non-coding RNA: TERRA. TERRA forms DNA/RNA hybrids at the telomeres which stimulate the generation of double-strand breaks, thereby facilitating DNA recombination-mediated VSG switching. Similarly, the telomere-binding proteins TRF and TIF2 also appear to play a role in suppressing DNA recombination at *T. brucei* telomeres, with their knockdown resulting in increased double-strand breaks and *VSG* switching [42,43]. However, it still remains to be determined if these proteins directly establish a repressed chromatin state at the *T .brucei* telomeres.

## How is the VSG switch mediated? Is there a link between antigenic variation and differentiation?

It has recently been proposed that antigenic variation is linked with differentiation in *T. brucei* [44,45]. Overexpression of a second ectopic VSG from an inducible promoter can result in transient silencing of the active ES proceeding upwards from the telomere. This transcriptional attenuation requires DOT1B-mediated histone methylation [36,46]. This provides evidence for the presence of a reversible chromatin-mediated silencing gradient extending up from the telomere end, and thereby having an impact on transcriptional elongation at the ES telomere. One possibility is that upon overexpression of the ectopic VSG, the trypanosome down-regulates the endogenous VSG in order to maintain homeostasis of VSG levels [47].

However, overexpression of the ectopic VSG also results in the generation of non-replicating ‘stumpy form’ parasites. These non-proliferative trypanosomes are pre-adapted to life in the tsetse fly and typically arise when cells sense that densities have risen above a given threshold. The authors therefore additionally postulate that this ES silencing is a ‘sensor’ that triggers stumpy differentiation and propose that this is initiated through silencing of *ESAG* genes located at the ES telomere [44].

However, an alternative interpretation for the results of Batram et al. [44] is that the overexpression of ectopic *VSG* (or knockdown of *ESAG*s) produces stress responses which trigger the generation of the stumpy form. One of the first events that occurs upon differentiation to the stumpy form is progressive attenuation of transcription of the active ES. This silencing gradient extends up from the telomere along the active ES, whereby the telomeric *VSG* is the first gene to be silenced [48]. The concurrent attenuation of *VSG* transcription at the active ES could therefore be a side effect of this stumpy form differentiation rather than directly causing it. This issue is discussed in ref. [45], but remains controversial. However, despite lack of clarity regarding the exact mechanistic details, these intriguing observations all argue that there is an intricate interplay between antigenic variation and trypanosome differentiation which still needs to be further unravelled.

In summary, new developments have highlighted the unprecedented complexity underlying antigenic variation in African trypanosomes. New experimental approaches are allowing us to tackle these issues at the whole-genome level using high-throughput sequencing of entire genomes or transcriptomes. Similarly, high-end proteomic approaches are allowing us to determine the interactomes of key players in the antigenic variation machinery. Whole-genome RNAi libraries are enabling identification of novel components using functional screens. Effective as these new approaches are, they are raising many more questions than answers regarding how African trypanosomes survive so well in the mammalian bloodstream.

## Outstanding questions

- How are chimeric *VSG* genes assembled? Is there a dedicated DNA recombination pathway for this?
- Where are chimeric *VSG*s assembled?
- What epigenetic factors mark silent vs. active *VSG* ESs?
- How do these components regulate the monoallelic expression of a single ES?
- How does antigenic variation have an impact on trypanosome differentiation and *vice versa*?

## Abbreviations

ES: expression site
ESB: expression site body
GPI: glycosylphosphatidylinositol
Pol I: polymerase I
VSG: variant surface glycoprotein
